# Empathy and resilience among Latin American medical students: examining associations with ecological resilience in a multicountry cross-sectional study

**DOI:** 10.1080/10872981.2026.2758968

**Published:** 2026-11-04

**Authors:** Víctor P. Díaz-Narváez, Rubén Eduardo Vázquez-García, Sara Huerta-González, Luz Marina Alonso Palacio, Juan Alberto Serrano Olvera, J. E. Rod, Anyel Bertel De la Hoz, Yolima Pertuz Meza, Eugenia S. González-Díaz, Nuvia Estrada-Méndez, Luis German Montero Saldaña, Yolanda Dávila Pontón, María Jorgelina Ulloque, María Gabriela Foscarini, María Guadalupe Silva-Vetri, Margarett Cuello Pérez, Jesús Alonso Cabrera, Aleyda I. Parra-Castillo, Mildred Amparo Sandoval, Lesbia Tirado Amador, Gary Caballero García, Karin Rondon-Payares, Zenen Antonio Carmona Meza, Javiera Santana, Isabela Fuentes Gonzalez, Estela Marcelo-Ascencio, José Gamarra-Moncayo

**Affiliations:** a Faculty of Dentistry, Andres Bello University, Santiago, Chile; b Faculty of Medicine, Universidad Veracruzana, Poza Rica, México; c Faculty of Nursing, Universidad Veracruzana, Poza Rica, México; d Department of Public Health, Universidad del Norte, Barranquilla, Colombia; e ABC Medical Centre, Mexico City CDMX, México; f Health Sciences Division, Universidad del Norte, Barranquilla, Colombia; g Faculty of Health Sciences, Universidad del Sinú Cartagena de Indias, Cartagena de Indias, Colombia; h Faculty of Nursing, Universidad Cooperativa de Colombia, Santa Marta, Colombia; i Faculty of Medicine, Universidad Central del Este, San Pedro de Macorís, República Dominicana; j Faculty of Dentistry, Evangelical University of El Salvador, San Salvador, El Salvador; k Faculty of Natural and Exact Sciences, Universidad Autónoma de Chiriquí, David, Chiriquí, Panamá; l Faculty of Law and Political and Social Sciences, Universidad de Cuenca, Cuenca, Ecuador; m Faculty of Health Sciences, Universidad Católica de Córdoba, Córdoba, Argentina; n Faculty of Health Sciences, Universidad Nacional Pedro Henríquez Ureña, Santo Domingo, República Dominicana; o Faculty of Health Sciences, Corporación Universitaria Rafael Núñez, Cartagena, Colombia; p Division of Basic Sciences, Universidad del Norte, Barranquilla, Colombia; q Faculty of Health Sciences, Universidad del Atlántico, Puerto Colombia, Colombia; r Research Department, Universidad Autónoma de Santa Ana, Santa Ana, El Salvador; s Faculty of Health Sciences, Universidad del Sinú Sectional Cartagena, Cartagena de Indias, Colombia; t Faculty of Health Sciences, Universidad del Magdalena, Santa Marta, Colombia; u Faculty of Medicine, University of Cartagena, Cartagena, Colombia; v Universidad de Concepción, Concepción, Chile; w Faculty of Medicine, Universidad de Cartagena, Bolívar, Colombia; x Faculty of Medicine, Universidad Católica Santo Toribio de Mogrovejo, Chiclayo, Perú

**Keywords:** Empathy, resilience, medical students, medical education, Latin America

## Abstract

**Background:**

Empathy is a core competency in medical education and underpins patient-centered care, yet empathy-related attitudes may decline during training, particularly under sustained academic and clinical stress. Resilience may help preserve empathic functioning, but its multidimensional associations with specific empathy domains remain insufficiently examined in large, culturally heterogeneous medical student samples.

**Objective:**

To examine associations between empathy dimensions and engineering, ecological, and adaptive resilience among medical students, and to evaluate the measurement properties of the study instruments across countries and gender.

**Methods:**

A cross-sectional multicountry study included 7,011 medical students from 11 universities in six Latin American countries. Structural equation modeling examined latent associations between empathy and resilience dimensions. Confirmatory factor analyses evaluated the factorial structure of the Jefferson Scale of Empathy–Health Professions Students and the Resilience Trait Scale, followed by measurement invariance testing by gender. Measurement models were evaluated before structural model estimation

**Results:**

Ecological resilience showed the strongest positive associations with empathy dimensions, whereas engineering and adaptive resilience showed smaller negative associations with selected empathy dimensions. In the total sample, both measurement models showed acceptable global fit, although some country-specific empathy models and the Walking in the Patient’s Shoes subscale showed psychometric limitations. Gender invariance was supported in most analyses.

**Conclusions:**

The findings suggest that resilience is a multidimensional correlate of empathy-related attitudes in medical students, with ecological resilience emerging as the dimension most consistently aligned with empathic functioning. Psychometric limitations should be considered when interpreting these associations. Educational strategies that support students’ capacity to maintain psychological stability under adversity may therefore warrant further evaluation as one component of broader efforts to sustain empathy and well-being during medical training. Because the data are cross-sectional, these implications should be tested in longitudinal and intervention studies.

## Introduction

Medical students constitute the future medical workforce and therefore represent an important population within medical education and health systems. Beyond technical competence, contemporary competency frameworks emphasize communication, collaboration, professionalism, and other interpersonal capacities relevant to patient care [1,2].

In the context of medical training, the Interprofessional Education Collaborative (IPEC) competencies recognize empathy, respect, and compassion as elements of professional practice and endorse person-centered care [1]. In clinical care, empathy can be conceptualized as a predominantly cognitive attribute involving the understanding of patients’ experiences, concerns, and perspectives, together with the capacity to communicate this understanding and an intention to help [3]. Within the conceptual framework of the Jefferson Scale of Empathy, empathy comprises three related dimensions: Perspective Adoption, defined as the cognitive capacity to understand the patient’s point of view; Compassionate Care, capturing the importance attributed to compassionate and emotionally responsive patient care; and Walking in the Patient’s Shoes, referring to the capacity to imaginatively place oneself in the patient’s position and appreciate the patient’s experience from that perspective. Empathy has been discussed in relation to patient-physician trust [4]. In practicing physicians, empathy scores have been positively associated with several patient-reported measures of communication and care experience [5].

The use of the term patient-centered care in this study reflects the Jefferson Scale’s focus on understanding patients’ perspectives and conveying that understanding in clinical encounters [3]. We situate this clinical focus within a broader person-centered orientation to professional education: the IPEC competencies emphasize individuals’ values and priorities, their participation in care decisions, and attention to determinants of health [1]. Thus, patient-centered terminology specifies the scope of the empathy construct examined; it does not imply that medical education is limited to illness care or that the study evaluates person-centered care in its entirety.

Several studies have reported a decline in empathy-related attitudes during medical training, particularly in more clinically demanding stages [6]. Although the mechanisms underlying this pattern remain unclear, academic demands, emotional stress, and exposure to clinically demanding stages have been proposed as contributing contextual factors [6–8].

Resilience has been conceptualized as a multidimensional psychosocial resource reflecting individuals’ capacity to adapt to stress and adversity [9,10]. In health professions students, resilience has been examined in relation to academic stress, psychological well-being, coping processes, and empathy, suggesting that resilience-related processes may be associated with professional attitudes and experiences during training [11–13].

In Maltby and colleagues’ three-facet model of resilience, engineering resilience refers to the capacity to recover or return to a previous state following adversity; ecological resilience captures the capacity to absorb or withstand adversity while maintaining psychological stability; and adaptive resilience is defined as the capacity to adjust and reorganize flexibly in response to changing or sustained adverse conditions [9,10].

In this study, empathy and resilience were considered multidimensional constructs whose specific dimensions may show different patterns of association. The distinction among engineering, ecological, and adaptive resilience provides a theoretical basis for examining these relationships separately rather than treating resilience as a single homogeneous construct [9,10].

Although previous research has explored links between resilience and empathy in healthcare professionals and students, findings remain inconsistent, and many studies have relied on small or single-institution samples. Moreover, limited evidence is available from large samples of medical students in low- and middle-income settings, where contextual and educational conditions may differ substantially.

Based on the theoretical frameworks described above and previous evidence in health professions students, it was hypothesized that empathy dimensions would show differential associations with engineering, ecological, and adaptive resilience [9,10,14]. The interpretation of relationships among multidimensional constructs requires attention to how those constructs are measured [15].

This study had two objectives: to examine latent associations between empathy dimensions and engineering, ecological, and adaptive resilience among medical students from 11 universities in six Latin American countries, and to evaluate the measurement properties of the study instruments across countries and gender.

## Materials and methods

### Design

A cross-sectional, observational, multicenter study was conducted among medical students enrolled at 11 universities across six Latin American countries: Argentina, Colombia, Ecuador, El Salvador, Panama, and the Dominican Republic.

### Participants

The study population comprised 10,012 medical students enrolled at 11 participating universities across six Latin American countries: Argentina (1 university), Colombia (4 universities), Ecuador (1 university), El Salvador (2 universities), Panama (1 university), and the Dominican Republic (2 universities). The final analytic sample consisted of 7,011 students, representing 70.0% of the total study population. Participants were recruited using a non-probabilistic, stratified convenience sampling strategy within each participating institution. The strata corresponded to the academic years represented in each medical program. Academic year was used exclusively as a stratification variable during recruitment; however, the individual-level distribution by academic year was not retained in the analytic dataset and therefore could not be reported retrospectively.

Eligible participants were students formally enrolled in the medical program at one of the participating institutions who voluntarily agreed to participate and provided the required informed consent. For students younger than 18 years, parental or legal guardian consent and student assent were required. Students were excluded from the analytic sample if questionnaires were incomplete or failed the predefined data-quality checks. Students who were absent during data collection, declined participation, or, when applicable, did not provide the required parental or guardian consent were not included in the study.

### Research instruments

Empathy was measured using the Jefferson Scale of Empathy–Health Professions Students version (JSE-HPS). The instrument comprises 20 items answered on a seven-point Likert-type scale ranging from 1 (strongly disagree) to 7 (strongly agree). After reverse scoring the negatively worded items, item scores are summed to obtain a total score ranging from 20 to 140, with higher scores indicating greater empathy-related attitudes. The scale comprises three dimensions: Perspective Adoption (PA; referred to as Perspective Taking in the original English version), Compassionate Care (CC), and Walking in the Patient’s Shoes (WIPS). In the measurement model specified for this study, PA comprised 10 items, CC eight items, and WIPS two items. Previous psychometric evaluations of the JSE-HPS include 3,712 nursing students from 11 Latin American schools [16] and 6,291 medical students from seven schools in Colombia, El Salvador, and the Dominican Republic, in whom the Spanish-language version showed *α* = 0.82, *ω* = 0.88, acceptable fit for the three-factor structure (CFI = 0.90), and factorial invariance by country and gender (ΔCFI < 0.01) [17]. In the 2011 study by Fields and colleagues involving 265 baccalaureate nursing students at a university in the southeastern United States, the JSE-HPS showed Cronbach’s *α* = 0.78 [18]. The JSE-HPS was used with permission from Thomas Jefferson University through the license held by the central project in Chile.

Resilience was measured using the 12-item Resilience Trait Scale (EEA), which corresponds to the five-response-category Resilient Systems Scales based on Maltby and colleagues’ three-facet resilience model [9,10]. The items are answered on a five-point Likert-type scale ranging from 1 (strongly disagree) to 5 (strongly agree). Item scores are summed to obtain a total score ranging from 12 to 60, with higher scores indicating greater resilience. The instrument assesses three dimensions—engineering, ecological, and adaptive resilience—each comprising four items [9,10].

The underlying three-facet resilience model was initially examined in psychology students and community adults in the United Kingdom [9]. The 12-item, five-response-category measure was subsequently evaluated in adult samples from the United States and university students in the United Kingdom [10]. More recent evidence from Latin America includes 397 dental students in El Salvador, in whom the resilience measure showed Cronbach’s *α* = 0.84 for the total score and *α* = 0.86, 0.80, and 0.75 for engineering, ecological, and adaptive resilience, respectively, with adequate fit for the three-factor structure (CFI = 0.96, RMSEA = 0.061, SRMR = 0.045) [14].

Both instruments were administered in Spanish. The JSE-HPS was administered in the Spanish-language version previously evaluated in medical students from Colombia, El Salvador, and the Dominican Republic; [17] therefore, no translation or country-specific review was performed. The EEA was originally developed in English [9]. In Argentina and El Salvador, the Spanish-language version administered to health sciences students in El Salvador was used [14]. In the Dominican Republic, Panama, Colombia, and Ecuador, where no locally validated Spanish-language version of the EEA was available at the time of study preparation, the EEA underwent a translation and back-translation procedure, followed by expert review using the Judges’ Criterion to ensure that item wording was clear and appropriate for local Spanish usage while preserving the original content. The translation and back-translation process was carried out by members of the academic team; no professional translators participated. The expert panel comprised five members: two full professors, two specialists in higher education pedagogy, and one social psychologist. Panel members were invited based on their academic and professional experience in areas related to instrument evaluation and validation; formal documentation of specific psychometric credentials was not required as a selection criterion. The translated version was reviewed using the Judges’ Criterion, and no changes were made to the original scale items. Following expert review, a pilot test was conducted with 20 medical students from different academic years to assess item comprehension. A detailed distribution by academic year was not retained. No additional comments or suggestions requiring changes to the EEA were reported, and no modifications were made to the scale items following the pilot test. The psychometric properties of both instruments were subsequently evaluated in the present multicountry sample.

### Data collection procedure

Data collection was conducted between August and November 2023 across the participating institutions. Within each participating institution, students were approached during scheduled academic activities according to the academic-year strata established for the sampling process. Before questionnaire administration, students were informed that their decision whether to participate would have no academic consequences and that they could withdraw without penalty. Students who agreed to participate provided the required informed consent; for participants younger than 18 years, parental or legal guardian consent and student assent were required.

The questionnaires were administered in person using a paper-based format in classroom settings under the supervision of faculty members who had been previously trained in the data-collection procedures. No identifying information was collected. After data collection, questionnaires that were incomplete or did not meet the predefined data-quality criteria were excluded from the analytic sample.

## Ethics

The research protocol and informed consent procedures were reviewed and approved by the Institutional Bioethics Committee of Universidad Andrés Bello (UNAB), Santiago, Chile (July 2022); record No. 020/2022, whose approval covered the multicenter study involving the participating universities in Latin America, as documented in the original protocol and supporting institutional agreements. The study was conducted in accordance with the ethical principles of the Declaration of Helsinki. Table S1 (additional file) presents the baseline characteristics of medical students by country and university. Of the 7,011 participants included in the analytic sample, 428 (6.1%) were younger than 18 years. For these participants, written informed consent was obtained from parents or legal guardians, together with the students’ assent. All participants provided consent for the use of their data for research and publication purposes. The database was irreversibly anonymized prior to analysis and is publicly available for research purposes through the Open Science Framework (OSF): https://osf.io/gd68r?view_only=3fa08db7d0454ad689546e28afa2f657.

### Data analysis

Descriptive analyses were first performed to characterize the sample and study variables. Categorical variables were summarized using absolute frequencies and percentages, whereas continuous scale scores were described using means and standard deviations. At the item level, means, standard deviations, skewness, and kurtosis were examined to characterize score distributions and departures from univariate normality. Gender differences in empathy and resilience scores were evaluated using independent-samples t tests. Effect sizes for between-gender comparisons were estimated using Cohen’s d and interpreted using the conventional benchmarks of approximately 0.20, 0.50, and 0.80 for small, medium, and large effects, respectively [19]. Statistical significance was set at *p* < 0.05.

The analyses are presented below according to the two study objectives. Measurement models were evaluated before estimation of the structural associations model.

### Objective 1 empathy and resilience associations

After evaluating the measurement properties of the instruments, a latent structural association model was specified to examine the theory-guided relationships between the empathy dimensions and engineering, ecological, and adaptive resilience, both in the total sample and by gender. For the combined structural model, robust maximum likelihood MLR was selected because all indicators had at least five ordered response categories; the items were treated as approximately continuous, and robust corrections were applied to address departures from multivariate normality [20]. Cross-sectional structural equation modeling (SEM) permits the examination of theoretically specified associations among latent constructs while accounting for measurement error; however, it does not establish temporal precedence or causal effects [15]. Accordingly, the estimated structural paths were interpreted as statistical associations rather than causal, predictive, or temporally ordered relationships. The same goodness-of-fit indices used for the confirmatory factor analysis were applied to evaluate structural model fit. For descriptive interpretation of standardized structural coefficients (*β*), absolute values < 0.20 were considered weak, values from 0.20 to 0.50 moderate, and values > 0.50 strong, following the criteria summarized by Domínguez-Lara [19]. These thresholds were used as interpretative guidelines rather than rigid indicators of substantive importance.

### Objective 2 measurement properties across countries and gender

The measurement models were evaluated using confirmatory factor analysis (CFA), first in the total sample and then separately by country to examine the psychometric functioning of the instruments across the participating settings. The choice of estimator was based on the measurement level and distributional characteristics of the items. The JSE-HPS uses seven ordered response categories; therefore, its items were treated as approximately continuous indicators, consistent with methodological evidence indicating that ordinal variables with six or seven categories can generally be analyzed using robust continuous-data methods under appropriate conditions [20]. Robust Maximum Likelihood (MLR) estimation was used for the JSE-HPS because it provides robust standard errors and a corrected model test in the presence of departures from multivariate normality, which was relevant given the skewness and kurtosis observed for some empathy items. In contrast, the EEA comprises five-category ordinal items and was modeled explicitly as an ordered categorical measure using the Weighted Least Squares Mean and Variance Adjusted (WLSMV) estimator, which is appropriate for CFA with ordinal indicators [20].

Model fit was evaluated using the Comparative Fit Index (CFI), Tucker–Lewis Index (TLI), Root Mean Square Error of Approximation (RMSEA), and Standardized Root Mean Square Residual (SRMR). Values of CFI and TLI > 0.90 and RMSEA and SRMR < 0.08 were considered indicative of acceptable model fit [21]. Following evaluation of the factorial structure, internal consistency was assessed using McDonald’s omega and Cronbach’s alpha coefficients, with values ≥ 0.70 considered acceptable [22].

Measurement invariance by gender was then examined sequentially through configural, metric, scalar, and strict invariance models in the total sample and within the country-specific samples. Changes in model fit were evaluated according to the criteria proposed by Rutkowski and Svetina, with ΔCFI ≤ 0.02 and ΔRMSEA ≤ 0.03 considered supportive of invariance [23]. Scalar invariance was considered the minimum level required to support meaningful comparisons of latent means across gender groups, whereas strict invariance additionally requires equality of residual variances. Country-specific analyses were conducted to evaluate measurement functioning rather than to estimate nationally representative differences between countries.

### Additional descriptive analyses

After evaluation of the measurement properties, percentile-based descriptive score categories were derived for descriptive purposes only, considering five ordinal levels for both scales. These categories were intended solely to summarize score distributions within the present sample and were not interpreted as clinical, diagnostic, pedagogical, or criterion-referenced thresholds.

All analyses were performed using JASP version 0.19.3 and R within the RStudio environment, employing the lavaan (version 0.6-19) and semTools (version 0.5-6) packages.

## Results

A total of 7,011 medical students participated in the study (66.8% female, *n* = 4,686; 33.2% male, *n* = 2,325), with a mean age of 20.7 years (SD = 2.8). Participants were from six Latin American countries and 11 medical schools: Argentina (*n* = 460), Colombia (*n* = 2,455), Ecuador (*n* = 543), El Salvador (*n* = 1,292), Panama (*n* = 692), and the Dominican Republic (*n* = 1,569). The sample included students from the academic years represented within the participating medical programs. A detailed distribution by academic year was not available in the analytic dataset.

### Empathy and resilience associations

A structural equation model was specified to examine the associations between empathy-related attitudes and resilience dimensions in the total sample and by gender. Standardized path coefficients showed positive and negative associations between specific empathy and resilience dimensions.

Table 1 presents the fit indices of the structural model examining relationships between empathy-related attitudes and resilience dimensions. The model showed an acceptable fit to the data in the total sample (CFI = 0.94; TLI = 0.94; RMSEA = 0.03 [90% CI: 0.03–0.03]; SRMR = 0.04) as well as in the gender-stratified analyses. Fit indices remained within recommended thresholds for both male students (CFI = 0.94, TLI = 0.94, RMSEA = 0.03, SRMR = 0.04) and female students (CFI = 0.94, TLI = 0.93, RMSEA = 0.03, SRMR = 0.04), indicating comparable model fit across gender subgroups.

**Table 1.** Model fit indices for the structural equation model examining associations between resilience and empathy.

Groups	$\chi^{2}$ (df)	*p*	CFI	TLI	RMSEA [90% CI]	SRMR
Male	1434.32 (449)	<.001	0.94	0.94	0.03 [0.03–0.03]	0.04
Female	2548.46 (449)	<.001	0.94	0.93	0.03 [0.03–0.03]	0.04
Total sample	3484.49 (449)	<.001	0.94	0.94	0.03 [0.03–0.03]	0.04

Note: CFI = Comparative Fit Index; TLI = Tucker-Lewis Index; RMSEA = Root Mean Square Error of Approximation; SRMR = Standardized Root Mean Square Residual Index.

### Structural model of relationships between resilience dimensions and empathy-related attitudes

Figure 1 presents the standardized path coefficients (*β*) of the structural model examining the relationships between empathy-related attitudes and resilience dimensions. Compassionate Care (CC) and Perspective Adoption (PA) showed weak negative associations (|β| < 0.20) with engineering (ENG) and adaptive (ADA) resilience, whereas ecological (ECO) resilience showed moderate positive associations (|β| = 0.20–0.50) with CC and PA. For Walking in the Patient’s Shoes (WIPS), a weak positive association was observed with ECO and a weak negative association with ADA. All reported paths were statistically significant (*p* < 0.05).

**Figure 1.**
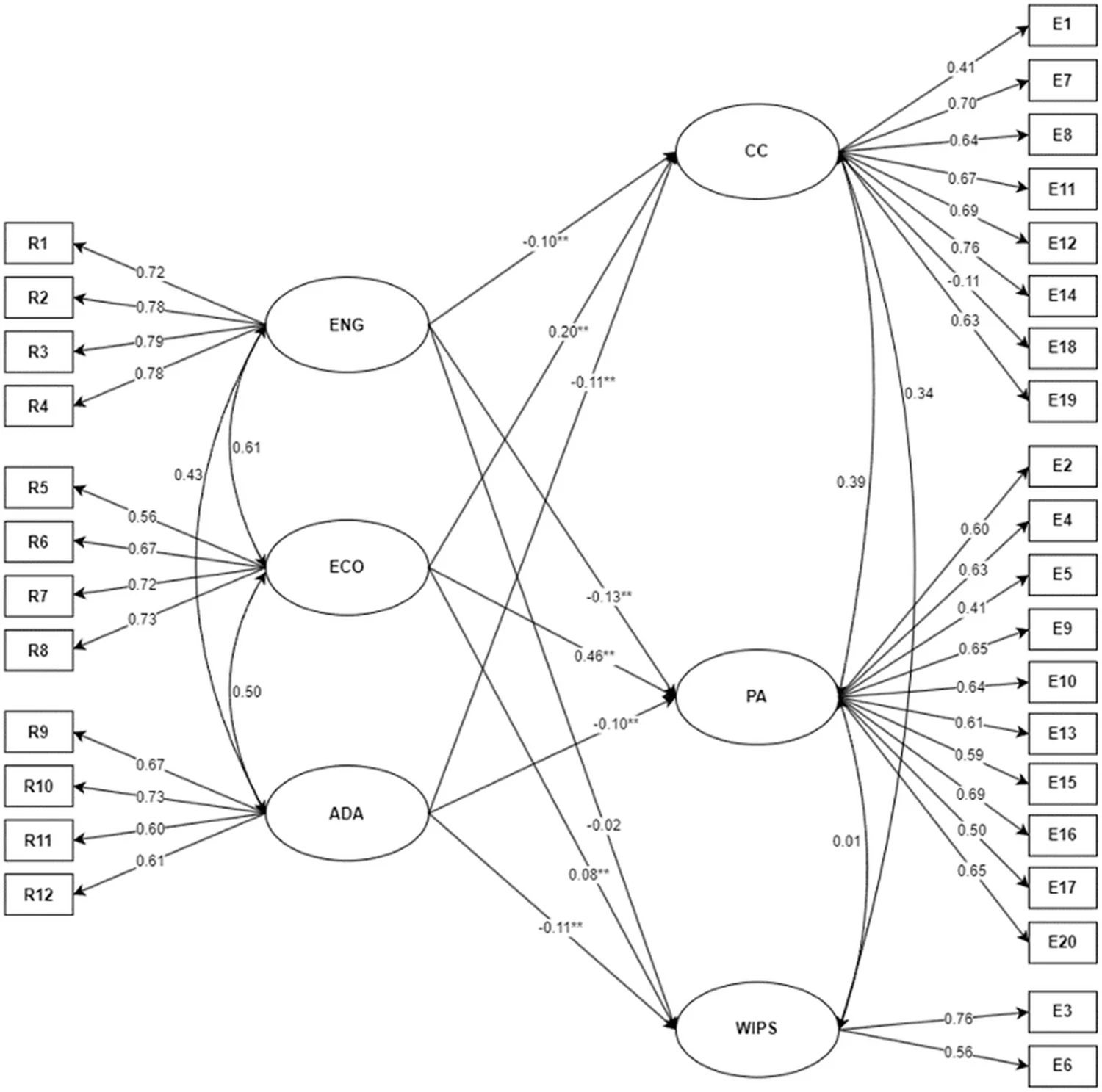
Structural equation model examining associations between resilience dimensions and empathy-related attitudes. Note: The standardized paths coefficients (*β*) between Resilience dimensions (ENG = Engineering, ECO = Ecological, ADA = Adaptive) and empathy dimensions (CC = Compassionate Care, PA = Perspective Adoption, WIPS = Walking in the Patient's Shoes) are shown. All displayed paths were statistically significant (*p* = < 0.05).

### Measurement properties across countries and gender

Table 2 presents the confirmatory factor analysis results for the JSE-HPS scale. In the total sample, the model showed acceptable fit indices (CFI = 0.94, TLI = 0.93, RMSEA = 0.05, SRMR = 0.04). In some countries, including Colombia (CFI = 0.85, TLI = 0.83), Ecuador (CFI = 0.89, TLI = 0.88), and Panama (CFI = 0.88, TLI = 0.87), CFI and TLI values were below the commonly recommended threshold of 0.90, while RMSEA and SRMR values remained within acceptable ranges.

**Table 2.** Model fit indices from confirmatory factor analysis of the empathy scale by country and in the total sample.

Country	$\chi^{2}$ (df)	*p*	CFI	TLI	RMSEA [90% CI]	SRMR
Argentina (*n* = 460)	293.97 (167)	<0.001	0.92	0.91	0.04 [0.04–0.05]	0.05
Colombia (*n* = 2455)	2037.97 (167)	<0.001	0.85	0.83	0.06 [0.06–0.07]	0.07
Ecuador (*n* = 543)	394.86 (167)	<0.001	0.89	0.88	0.05 [0.05–0.06]	0.07
El Salvador (*n* = 1292)	381.97 (167)	<0.001	0.94	0.94	0.03 [0.03–0.04]	0.04
Panama (*n* = 692)	389.92 (167)	<0.001	0.88	0.87	0.05 [0.04–0.06]	0.05
Dominican Republic (*n* = 1569)	863.09 (167)	<0.001	0.90	0.89	0.05 [0.05–0.06]	0.06
Total Sample (*n* = 7011)	2103.12 (167)	<0.001	0.94	0.93	0.05 [0.04–0.05]	0.04

Note: CFI = Comparative Fit Index; TLI = Tucker-Lewis Index; RMSEA = Root Mean Square Error of Approximation; SRMR = Standardized Root Mean Square Residual.

Table 3 presents the CFA results for the resilience scale across countries and in the total sample. CFI and TLI values ranged from 0.95 to 0.99, and RMSEA values ranged from 0.04 to 0.09. Overall, the proposed factor structure showed acceptable fit in most samples, although RMSEA exceeded or reached the predefined criterion in Argentina, Panama and Colombia.

**Table 3.** Model fit indices from confirmatory factor analysis of the resilience scale by country and in the total sample.

Country	$\chi^{2}$ (df)	*p*	CFI	TLI	RMSEA [90% CI]	SRMR
Argentina (*n* = 460)	240.49 (51)	<0.001	0.98	0.97	0.09 [0.08–0.10]	0.05
Colombia (*n* = 2455)	761.21 (51)	<0.001	0.98	0.98	0.08 [0.07–0.08]	0.03
Ecuador (*n* = 543)	155.97 (51)	<0.001	0.99	0.99	0.06 [0.05–0.07]	0.02
El Salvador (*n* = 1292)	172.75 (51)	<0.001	0.97	0.96	0.04 [0.04–0.05]	0.04
Panama (*n* = 692)	348.35 (51)	<0.001	0.96	0.95	0.09 [0.08–0.10]	0.05
Dominican Republic (*n* = 1569)	545.41 (51)	<0.001	0.97	0.97	0.07 [0.07–0.09]	0.05
Total Sample (*n* = 7011)	1611.35 (51)	<0.001	0.98	0.97	0.07 [0.06–0.07]	0.03

Note: CFI = Comparative Fit Index; TLI = Tucker-Lewis Index; RMSEA = Root Mean Square Error of Approximation; SRMR = Standardized Root Mean Square Residual.

Table 4 presents the internal consistency reliability coefficients for the empathy and resilience scales by country and in the total sample. Compassionate Care showed reliability coefficients below the reference threshold in Argentina and El Salvador. In the total sample, WIPS showed lower internal consistency (*α* = 0.60; *ω* = 0.61), with substantial variability across countries. In El Salvador, the three resilience dimensions also showed reliability coefficients below the reference threshold (*α* = 0.59–0.64; *ω* = 0.60–0.65).

**Table 4.** Internal consistency reliability (Cronbach's alpha and McDonald’s omega) for the empathy and resilience scales by country and total sample.

Country	Index	Empathy			Resilience		
		CC	PA	WIPS	ENG	ECO	ADA
Argentina	α	0.66	0.78	0.64	0.9	0.76	0.81
	ω	0.66	0.77	0.71	0.93	0.77	0.82
Colombia	α	0.79	0.83	0.66	0.89	0.79	0.76
	ω	0.80	0.83	0.66	0.92	0.81	0.77
Ecuador	α	0.82	0.89	0.44	0.93	0.87	0.88
	ω	0.82	0.89	0.73	0.94	0.87	0.89
El Salvador	α	0.57	0.82	0.38	0.63	0.64	0.59
	ω	0.53	0.82	0.38	0.64	0.65	0.60
Panama	α	0.72	0.75	0.65	0.86	0.77	0.77
	ω	0.74	0.75	0.67	0.90	0.78	0.79
Dominican Republic	α	0.76	0.85	0.66	0.87	0.75	0.74
	ω	0.76	0.85	0.67	0.90	0.75	0.76
Total sample	α	0.78	0.84	0.60	0.85	0.77	0.75
	ω	0.79	0.84	0.61	0.87	0.77	0.76

Note: CC = Compassionate Care, PA = Perspective Adoption; WIPS = Walking in the Patient ´ s Shoes; ENG = Engineering resilience; ECO = Ecological resilience; ADA = Adaptive resilience. *α* = Cronbach ´ s alpha; *ω* = McDonald’s omega.

Table 5 presents the measurement invariance results by gender for the empathy scale. Configural, metric, and scalar invariance were supported across countries and in the total sample, except in El Salvador.

**Table 5.** Measurement invariance of the empathy scale across gender by country and in the total sample.

Country^a^	Level	$\chi^{2}$ (df)	*p*	CFI	ΔCFI	RMSEA	ΔRMSEA	SRMR
Argentina	Configural	502.66 (336)	<0.001	0.880		0.047		0.060
	Metric	535.53 (351)	<0.001	0.869	−0.011	0.048	0.001	0.067
	Scalar	567.39 (368)	<0.001	0.858	−0.011	0.049	0.001	0.070
	Strict	616.29 (388)	<0.001	0.838	−0.02	0.051	0.002	0.078
Colombia	Configural	2231.64 (334)	<0.001	0.838		0.068		0.066
	Metric	2269.86 (351)	<0.001	0.836	−0.002	0.067	−0.001	0.068
	Scalar	2311.64 (368)	<0.001	0.834	−0.002	0.066	−0.001	0.068
	Strict	2263.42 (388)	<0.001	0.840	0.006	0.063	−0.003	0.068
Ecuador	Configural	590.88 (334)	<0.001	0.877		0.053		0.074
	Metric	602.52 (351)	<0.001	0.879	0.002	0.051	−0.002	0.076
	Scalar	613.80 (368)	<0.001	0.882	0.003	0.050	−0.001	0.076
	Strict	614.55 (388)	<0.001	0.891	0.009	0.046	−0.004	0.076
Panama	Configural	578.33 (334)	<0.001	0.874		0.046		0.056
	Metric	607.55 (351)	<0.001	0.867	−0.007	0.046	0	0.061
	Scalar	639.97 (368)	<0.001	0.859	−0.008	0.046	0	0.062
	Strict	665.04 (388)	<0.001	0.857	−0.002	0.045	−0.001	0.066
Dominican Republic	Configural	1092.80 (334)	<0.001	0.890		0.054		0.056
	Metric	1097.86 (351)	<0.001	0.892	0.002	0.052	−0.002	0.057
	Scalar	1121.87 (368)	<0.001	0.891	−0.001	0.051	−0.001	0.058
	Strict	1077.63 (388)	<0.001	0.900	0.009	0.048	−0.003	0.058
Total Sample	Configural	2325.87 (334)	<0.001	0.931		0.041		0.043
	Metric	2374.59 (351)	<0.001	0.930	−0.001	0.041	0	0.045
	Scalar	2439.36 (368)	<0.001	0.928	−0.002	0.040	−0.001	0.045
	Strict	2437.32 (388)	<0.001	0.929	0.001	0.039	−0.001	0.045

Note: Gender measurement invariance could not be fully established in the El Salvador sample.

Table S2 (additional file) presents the gender-based measurement invariance results for the resilience scale across countries. Measurement invariance was supported in the evaluable country-specific samples and in the total sample. In the Dominican Republic, the full sequence of gender invariance models could not be evaluated because one response category was absent for one resilience item in the male subgroup, preventing estimation of the corresponding ordinal thresholds.

### Additional descriptive analyses

Table S3 (additional file) presents the item level descriptive statistics for the empathy and resilience scales. For the JSE-HPS, item means ranged from 3.19 to 6.24, with SDs ranging from 1.38-2.24, skewness values from −2.22 to 0.46 and kurtosis values from −1.39 to 4.47. For the EEA, item means ranged from 3.18 to 4.14, with SDs ranging from 0.97 - 1.22, skewness values from −1.17 to −0.11 and kurtosis values from −0.80 and 1.04.

It was observed that some items of the empathy scale (items 2, 4, and 20) presented skewness and/or kurtosis indices exceeding the reference threshold adopted in this study (|2|), indicating deviations from the reference thresholds for univariate normality [24].

Table 6 shows gender differences in empathy and resilience scores. Female students reported higher mean empathy scores (M = 103.99, SD = 17.02) than male students (M = 102.39, SD = 16.67), t (7009) = −3.76, *p* < 0.01, d = 0.095. In contrast, male students reported higher mean resilience scores (M = 44.49, SD = 8.13) than female students (M = 42.46, SD = 7.93), t (7009) = 10.01, *p* < 0.001, d = 0.254.

**Table 6.** Mean scores (SD) for empathy and resilience dimensions by gender.

	Female (*n* = 4686)		Male (*n* = 2325)		Total (*n* = 7011)				
Dimensions	M	SD	M	SD	M	SD	t (df)	*p*	*d*
Compassionate Care	37.84	10.46	37.21	10.09	37.63	10.34	−2.39 (7009)	0.017	0.061
Perspective Adoption	58.18	9.92	57.33	9.79	57.90	9.89	−3.38 (7009)	<0.001	0.086
Walking in the Patient’s Shoes	7.98	2.88	7.84	2.89	7.93	2.88	−1.88 (7009)	0.060	0.048
Empathy	103.99	17.02	102.39	16.67	103.46	16.92	−3.76 (7009)	<0.001	0.095
Engineering Resilience	13.44	3.70	14.51	3.66	13.79	3.72	11.39 (7009)	<0.001	0.289
Ecological Resilience	15.77	3.07	16.04	3.04	15.86	3.06	3.46 (7009)	<0.001	0.088
Adaptive Resilience	13.26	3.60	13.95	3.57	13.49	3.61	7.64 (7009)	<0.001	0.194
Resilience	42.46	7.93	44.49	8.13	43.14	8.05	10.01 (7009)	<0.001	0.254

Note: M = Mean; SD = standard deviation; d = Cohen's d. A *p* value < 0.05 was considered statistically significant.

Statistically significant gender differences were also observed across most dimensions of both instruments, except for Walking in the Patient’s Shoes (*p* > 0.05). However, the magnitude of these differences was consistently small (Cohen’s d < 0.30) [19]. In particular, the difference in total empathy scores was negligible in magnitude (d = 0.095), whereas the difference in total resilience scores was small (d = 0.254). Thus, despite statistical significance, the observed gender differences should not be interpreted as indicating substantial practical differences between male and female students.

Percentile-based descriptive score categories were derived for the empathy and resilience scales using five ordinal levels. These categories were used solely to summarize score distributions within the present sample and are presented in Table S4 (additional file).

## Discussion

The present multinational study had two objectives: to examine associations between empathy dimensions and engineering, ecological, and adaptive resilience among medical students from six Latin American countries, and to evaluate the measurement properties of the study instruments across countries and gender.

Previous studies have described declines in empathy-related attitudes during medical training, often referred to as empathic erosion, particularly in clinically demanding stages in which academic demands and emotional stress have been proposed as contributing factors [6–8]. Within this framework, the positive association observed between ecological resilience—the capacity to absorb adversity while maintaining psychological stability—and empathy dimensions is of particular interest, because it identifies a resilience process that coexists with higher empathy-related attitudes in a population exposed to the conditions associated with empathic erosion.

### Structural association model between empathy and resilience

The SEM showed acceptable global fit in the total sample. Given the heterogeneous measurement performance of the empathy scale, the structural findings should be interpreted as overall patterns within the present sample rather than as equally robust associations across all empathy dimensions and country contexts.

### Interpretation of the associations between empathy and resilience dimensions

The results suggest that resilience dimensions are differentially associated with the empathy dimensions. In particular, the ecological dimension showed small to moderate positive associations with empathy, while the engineering and adaptive dimensions showed negative associations of smaller magnitude.

In the present study, ecological resilience showed positive associations with the empathy dimensions examined.

Engineering resilience, understood as the ability to return to a previous state after a negative event [10], showed negative associations with the Compassionate Care (CC) and Perspective Adoption (PA) dimensions of empathy.

Because the data are cross-sectional, the observed associations cannot establish temporal order, directionality, causal effects, or underlying mechanisms. Accordingly, the positive association between ecological resilience and empathy should not be interpreted as evidence that ecological resilience protects against empathic erosion; likewise, the negative associations involving engineering and adaptive resilience do not establish that these dimensions diminish empathic functioning. The findings describe patterns of covariation in the present sample.

By examining a large, diverse, multinational cohort, this study extends existing evidence on the associations between resilience and empathy-related attitudes in health professions students [25,26]. The observed patterns identify resilience-related dimensions as variables that warrant further investigation in longitudinal studies examining empathy trajectories during medical training. Future research should determine whether changes in specific resilience processes are prospectively associated with changes in empathy before educational interventions targeting these constructs can be recommended.

To address the second objective, confirmatory factor analysis, reliability assessment, and measurement invariance testing were used to evaluate the study instruments, in line with current methodological recommendations for latent-variable models in cross-sectional designs [15].

The psychometric findings were not uniform across instruments, countries, or empathy dimensions. Although the JSE-HPS showed acceptable global fit in the total sample, lower CFI and TLI values were observed in Colombia, Ecuador, and Panama, and the reliability of some empathy dimensions was below the predefined criterion. Consequently, subsequent findings involving specific empathy dimensions should be interpreted with greater caution than the overall model fit might otherwise suggest, especially when considering their applicability across different country contexts.

Women had higher empathy-related scores, whereas men had higher resilience scores; however, all gender differences were small or negligible (Cohen’s d < 0.30), indicating limited practical significance and providing no basis for gender-specific educational strategies. Similar patterns have been reported previously, although findings in Latin America remain heterogeneous [17,27–31]. Psychological distress and mental health among medical students, examined in related literature [32–36], were not assessed in the present study.

Measurement invariance analyses supported gender-based comparisons in most samples. However, invariance does not imply uniformly adequate reliability or model fit in every subgroup. Therefore, gender comparisons should be interpreted cautiously in light of the heterogeneous psychometric performance across countries and dimensions. These results should not be taken as evidence of complete psychometric equivalence across all participating contexts.

In some countries, the reliability of specific empathy dimensions, particularly the WIPS subscale, did not reach the expected threshold. This finding represents a genuine psychometric limitation that may reflect the difficulty of capturing this dimension consistently across educational and cultural contexts. Future studies should examine the functioning of these items in greater detail, including item-level analyses, differential item functioning, and qualitative evidence of item comprehension across countries.

The percentile-based categories are descriptive, sample-dependent references derived from the present cohort and should not be interpreted as validated cut-off points or individual-level thresholds. Their use to assess change over time or evaluate interventions requires independent longitudinal validation [16,37,38].

### Implications for educational policy and preventive strategies

The findings of this study suggest that resilience and empathy may be relevant constructs for further investigation within health professions education. Given the cross-sectional design, the present results should be considered hypothesis-generating and do not provide a basis for recommending specific educational policies, screening procedures, or interventions. From an institutional perspective, the results may inform future research on how empathy and resilience are distributed among medical students. Any educational or preventive strategy should rely on multiple sources of evidence, including continuous scale scores, academic and clinical indicators, student well-being measures, and contextual information from training environments.

Furthermore, although statistically significant gender differences were observed, their small effect sizes do not provide sufficient evidence to justify gender-specific educational policies on the basis of the present findings alone. Future research should examine whether gender interacts with educational, psychosocial, and contextual factors in ways that are both statistically and practically meaningful before specific gender-tailored strategies are recommended.

### Study limitations

One of the main limitations concerns the sampling strategy and the scope of inference. Participants were recruited using a non-probabilistic, stratified convenience sampling strategy from 11 universities in six Latin American countries. Although the sample was large and represented a substantial proportion of the student population at the participating institutions, neither the institutions nor the participants were selected to produce nationally representative samples. Accordingly, the findings should not be interpreted as country-level estimates or generalized to all medical students within the participating countries or across Latin America. The multicenter design increases the diversity of educational contexts represented in the study but does not, by itself, confer population representativeness.

Furthermore, although the models accounted for gender and country, other relevant contextual and individual factors—such as academic workload, psychological distress, burnout, or institutional characteristics—were not included and may act as unmeasured confounders.

Another relevant limitation concerns the psychometric performance of the empathy scale. Although the total-sample CFA showed acceptable fit, some country-specific fit indices were below conventional thresholds, and the WIPS dimension showed lower internal consistency. These findings limit the precision and generalizability of dimension-specific interpretations. Finally, because the study was cross-sectional, temporal ordering between resilience and empathy cannot be established. Therefore, the structural paths should be interpreted exclusively as contemporaneous statistical associations and not as evidence that resilience influences, predicts, protects against, or produces changes in empathy. Longitudinal and intervention studies are required to establish the direction and temporal stability of these relationships.

## Conclusions

This study identified differential cross-sectional associations between empathy and resilience dimensions among medical students from the participating universities, with ecological resilience showing a consistent positive pattern of association with empathy dimensions. The instruments showed acceptable global fit in the total sample, but heterogeneous country-specific performance and subscale reliability warrant caution. These findings describe associations within the participating institutions rather than causal relationships or nationally representative estimates.

Overall, the findings support examining resilience as a multidimensional construct in relation to empathy-related attitudes. Longitudinal and intervention studies are needed to determine whether resilience-related processes are associated with changes in empathy during medical training.

## Supplementary Material

Table S1.docx Table S1.docx

Table S4.docx Table S4.docx

Table S2.docx Table S2.docx

Table S3.docx Table S3.docx

## Data Availability

The datasets generated and analyzed during the current study are publicly available in the Open Science Framework (OSF) repository at: https://osf.io/gd68r?view_only=3fa08db7d0454ad689546e28afa2f657.

## Notes

All authors read and approved the final version of the manuscript and agree to be accountable for all aspects of the work.
