# Supplementary material for: Empathy and resilience among Latin American medical students: examining associations with ecological resilience in a multicountry cross-sectional study
Source: Med Educ Online. 2026 Nov 4;31(1):2758968. doi: 10.1080/10872981.2026.2758968 (PMC13757817; doi:10.1080/10872981.2026.2758968)
Supplement: Supplementary file 1 [file ZMEO_A_2758968_SM3617.docx]

**Table S1. Baseline characteristics of medical students by country and university**

| **Country** | **University** | **n** | **Age, mean (SD)** | **Female, n (%)** | **Male n (%)** | **< 18 years, n (%)** |
| --- | --- | --- | --- | --- | --- | --- |
| Argentina | U3 | 460 | 21.4 (2.6) | 341 (74.1) | 119 (25.9) | 0 (0.0) |
| Colombia | U5 | 437 | 20.5 (2.9) | 315 (72.1) | 122 (27.9) | 44 (10.1) |
| Colombia | U6 | 414 | 20.1 (2.5) | 222 (53.6) | 192 (46.4) | 54 (13.0) |
| Colombia | U8 | 604 | 19.1 (1.8) | 340 (56.3) | 264 (43.7) | 110 (18.2) |
| Colombia | U9 | 1000 | 20.2 (2.7) | 600 (60.0) | 400 (40.0) | 98 (9.8) |
| Ecuador | U7 | 543 | 20.3 (2.1) | 376 (69.2) | 167 (30.8) | 5 (0.9) |
| El Salvador | U2 | 465 | 21.3 (3.1) | 312 (67.1) | 153 (32.9) | 35 (7.5) |
| El Salvador | U10 | 827 | 21.2 (2.6) | 553 (66.9) | 274 (33.1) | 57 (6.9) |
| Panama | U1 | 692 | 21.6 (2.9) | 441 (63.7) | 251 (36.3) | 0 (0.0) |
| Dominican Republic | U4 | 930 | 20.5 (2.3) | 728 (78.3) | 202 (21.7) | 22 (2.4) |
| Dominican Republic | U11 | 639 | 22.5 (2.9) | 458 (71.7) | 181 (28.3) | 3 (0.5) |

***Note***: Academic year was used as a stratification variable during recruitment, but the individual-level academic-year variable was not retained in the analytical dataset; therefore, a detailed distribution by academic year cannot be reported retrospectively.
