# Supplementary material for: Empathy and resilience among Latin American medical students: examining associations with ecological resilience in a multicountry cross-sectional study
Source: Med Educ Online. 2026 Nov 4;31(1):2758968. doi: 10.1080/10872981.2026.2758968 (PMC13757817; doi:10.1080/10872981.2026.2758968)
Supplement: Supplementary file 2 [file ZMEO_A_2758968_SM3618.docx]

**Table S4. Percentile-based descriptive score categories for empathy and resilience dimensions in the total sample**

| **PC** | **CC** | **PA** | **WIPS** | **EMP** | **ENG** | **ECO** | **ADA** | **RES** | **Level** |
| --- | --- | --- | --- | --- | --- | --- | --- | --- | --- |
| 99 | 54 | 70 | 14 | 134 | 20 | 20 | 20 | 60 | Very high |
| 90 | 50 | 69 | 12 | 125 | 19 | 19 | 18 | 54 |  |
| 80 | 47 | 67 | 11 | 119 | 17 | 18 | 17 | 50 | High |
| 70 | 44 | 64 | 10 | 115 | 16 | 17 | 16 | 47 |  |
| 60 | 42 | 63 | 9 | 110 | 15 | 16 | 15 | 45 | Average |
| 50 | 39 | 60 | 8 | 105 | 14 | 15 | 14 | 43 |  |
| 41 | 36 | 58 | 7 | 100 | 13 | 14 | 13 | 41 |  |
| 30 | 33 | 55 | 6 | 93 | 12 | 13 | 12 | 39 | Low |
| 20 | 29 | 50 | 5 | 86 | 11 | 12 | 11 | 36 |  |
| 10 | 22 | 43 | 4 | 80 | 9 | 11 | 9 | 33 | Very low |
| 1 | 12 | 29 | 2 | 67 | 4 | 7 | 4 | 24 |  |

***Note****.* PC= Percentile; CC= Compassionate Care; PA= Perspective Adoption; WIPS= Walking in the Patient´s Shoes; EMP= Empathy; RES= Resilience; ENG= Engineering resilience; ECO= Ecological resilience; ADA= Adaptive resilience. Percentile-based categories were derived from the empirical distribution of the total sample for descriptive purpose only. They are sample-dependent and should not be interpreted as clinical, diagnostic, pedagogical, or criterion-referenced thresholds.
