# Supplementary material for: Empathy and resilience among Latin American medical students: examining associations with ecological resilience in a multicountry cross-sectional study
Source: Med Educ Online. 2026 Nov 4;31(1):2758968. doi: 10.1080/10872981.2026.2758968 (PMC13757817; doi:10.1080/10872981.2026.2758968)
Supplement: Supplementary file 3 [file ZMEO_A_2758968_SM3619.docx]

**Table S2. Gender-based measurement invariance of the resilience scale across countries**

| **Country ^a^** | **Level** | **X^2^ (df)** | **p** | **CFI** | **ΔCFI** | **RMSEA** | **ΔRMSEA** | **SRMR** |
| --- | --- | --- | --- | --- | --- | --- | --- | --- |
| Argentina | Configural | 281.04 (102) | <0.001 | 0.977 |  | 0.088 |  | 0.059 |
|  | Metric | 283.59 (111) | <0.001 | 0.978 | 0.001 | 0.082 | -0.006 | 0.063 |
|  | Scalar | 335.53 (144) | <0.001 | 0.975 | -0.003 | 0.076 | -0.006 | 0.061 |
|  | Strict | 335.53 (144) | <0.001 | 0.975 | 0.000 | 0.076 | 0.000 | 0.061 |
| Colombia | Configural | 815.29 (102) | <0.001 | 0.981 |  | 0.076 |  | 0.036 |
|  | Metric | 708.37 (111) | <0.001 | 0.984 | 0.003 | 0.066 | -0.010 | 0.038 |
|  | Scalar | 784.40 (144) | <0.001 | 0.983 | -0.001 | 0.060 | -0.006 | 0.037 |
|  | Strict | 784.40 (144) | <0.001 | 0.983 | 0.000 | 0.060 | 0.000 | 0.037 |
| Ecuador | Configural | 237.74 (102) | <0.001 | 0.991 |  | 0.070 |  | 0.029 |
|  | Metric | 247.53 (111) | <0.001 | 0.991 | 0.000 | 0.067 | -0.003 | 0.032 |
|  | Scalar | 244.42 (144) | <0.001 | 0.993 | 0.002 | 0.051 | -0.016 | 0.029 |
|  | Strict | 244.42 (144) | <0.001 | 0.993 | 0.000 | 0.051 | 0.000 | 0.029 |
| El Salvador | Configural | 228.15 (102) | <0.001 | 0.963 |  | 0.044 |  | 0.040 |
|  | Metric | 244.06 (111) | <0.001 | 0.961 | -0.002 | 0.043 | -0.001 | 0.042 |
|  | Scalar | 259.25 (144) | <0.001 | 0.966 | 0.005 | 0.035 | -0.008 | 0.041 |
|  | Strict | 259.25 (144) | <0.001 | 0.966 | 0.000 | 0.035 | 0.000 | 0.041 |
| Panamá | Configural | 404.55 (102) | <0.001 | 0.960 |  | 0.093 |  | 0.058 |
|  | Metric | 393.83 (111) | <0.001 | 0.962 | 0.002 | 0.086 | -0.007 | 0.062 |
|  | Scalar | 426.12 (144) | <0.001 | 0.962 | 0.000 | 0.075 | -0.011 | 0.059 |
|  | Strict | 426.12 (144) | <0.001 | 0.962 | 0.000 | 0.075 | 0.000 | 0.059 |
| Total Sample | Configural | 1616.55 (102) | <0.001 | 0.978 |  | 0.065 |  | 0.033 |
|  | Metric | 1447.41 (111) | <0.001 | 0.981 | 0.003 | 0.059 | -0.006 | 0.034 |
|  | Scalar | 1570.61 (144) | <0.001 | 0.979 | -0.002 | 0.053 | -0.006 | 0.033 |
|  | Strict | 1570.61 (144) | <0.001 | 0.979 | 0.000 | 0.053 | 0.000 | 0.033 |

***Note.*** Measurement invariance was evaluated using changes in CFI (ΔCFI ≤ 0.02) and RMSEA (ΔRMSEA ≤0.03). Gender measurement invariance could not be fully evaluated in the Dominican Republic because one response category was absent for one resilience item in the male subgroup, preventing estimation of the corresponding ordinal thresholds.
