# Supplementary material for: Empathy and resilience among Latin American medical students: examining associations with ecological resilience in a multicountry cross-sectional study
Source: Med Educ Online. 2026 Nov 4;31(1):2758968. doi: 10.1080/10872981.2026.2758968 (PMC13757817; doi:10.1080/10872981.2026.2758968)
Supplement: Supplementary file 4 [file ZMEO_A_2758968_SM3620.docx]

**Table S3. Item-level descriptive statistics for the empathy and resilience scales**

| **Empathy**  **Scale** | **M** | **σ** | **g1** | **g2** | **Item** | **M** | **σ** | **g1** | **g2** |
| --- | --- | --- | --- | --- | --- | --- | --- | --- | --- |
| 1 | 4.44 | 2.24 | -0.29 | -1.39 | 11 | 4.68 | 2.00 | -0.47 | -1.01 |
| 2 | 6.24 | 1.40 | -2.22 | 4.47 | 12 | 4.84 | 2.15 | -0.56 | -1.12 |
| 3 | 4.20 | 1.67 | -0.09 | -0.73 | 13 | 5.72 | 1.63 | -1.29 | 0.83 |
| 4 | 6.18 | 1.38 | -1.94 | 3.32 | 14 | 5.15 | 2.04 | -0.81 | -0.70 |
| 5 | 5.29 | 1.73 | -0.86 | -0.19 | 15 | 5.75 | 1.61 | -1.27 | 0.83 |
| 6 | 3.74 | 1.74 | 0.12 | -0.85 | 16 | 5.83 | 1.45 | -1.29 | 1.15 |
| 7 | 5.38 | 2.16 | -1.01 | -0.54 | 17 | 5.20 | 1.67 | -0.70 | -0.34 |
| 8 | 4.75 | 2.08 | -0.48 | -1.13 | 18 | 3.19 | 1.87 | 0.46 | -0.85 |
| 9 | 5.74 | 1.55 | -1.26 | 0.90 | 19 | 5.19 | 2.02 | -0.79 | -0.70 |
| 10 | 5.86 | 1.46 | -1.38 | 1.39 | 20 | 6.09 | 1.47 | -1.77 | 2.54 |
| **Resilience**  **Scale** | | | | | | | | | |
| 1 | 3.55 | 1.12 | -0.53 | -0.44 | 7 | 3.90 | 1.02 | -0.85 | 0.32 |
| 2 | 3.38 | 1.12 | -0.29 | -0.65 | 8 | 3.99 | 0.97 | -0.91 | 0.60 |
| 3 | 3.47 | 1.12 | -0.38 | -0.62 | 9 | 3.52 | 1.16 | -0.43 | -0.53 |
| 4 | 3.39 | 1.12 | -0.27 | -0.71 | 10 | 3.18 | 1.19 | -0.11 | -0.78 |
| 5 | 4.14 | 0.98 | -1.17 | 1.04 | 11 | 3.31 | 1.20 | -0.27 | -0.80 |
| 6 | 3.83 | 1.03 | -0.70 | -0.05 | 12 | 3.49 | 1.22 | -0.46 | -0.69 |

***Note****.* M= mean, σ= Standard deviation; g1= skewness; g2= Kurtosis.
